# Optimizing the design of a contraceptive microarray patch: a discrete choice experiment on women’s preferences in India and Nigeria

**DOI:** 10.1186/s12978-021-01113-y

**Published:** 2021-03-22

**Authors:** Rebecca L. Callahan, Aurélie Brunie, Victoria Lebrun, Mario Chen, Christine L. Godwin, Kanchan Lakhwani, Funmilola M. OlaOlorun

**Affiliations:** 1 FHI 360, Durham, NC USA; 2 FHI 360, Washington, DC USA; 3 Centre for Operations Research and Training (CORT), Baroda, Gujarat India; 4 College of Medicine, University of Ibadan, Ibadan, Nigeria

**Keywords:** Microarray patch, Contraception, User preferences, Discrete choice experiment, Nigeria, India

## Abstract

**Background:**

Efforts are underway to develop an easy-to-use contraceptive microarray patch (MAP) that could expand the range of self-administrable methods. This paper presents results from a discrete choice experiment (DCE) designed to support optimal product design.

**Methods:**

We conducted a DCE survey of users and non-users of contraception in New Delhi, India (496 women) and Ibadan, Nigeria (two versions with 530 and 416 women, respectively) to assess stated preferences for up to six potential product attributes: effect on menstruation, duration of effectiveness, application pain, location, rash after application, and patch size. We estimated Hierarchical Bayes coefficients (utilities) for each attribute level and ran simulations comparing women’s preferences for hypothetical MAPs with varying attribute combinations.

**Results:**

The most important attributes of the MAP were potential for menstrual side effects (55% of preferences in India and 42% in Nigeria) and duration (13% of preferences in India and 24% in Nigeria). Women preferred a regular period over an irregular or no period, and a six-month duration to three or one month. Simulations show that the most ideal design would be a small patch, providing 6 months of protection, that would involve no pain on administration, result in a one-day rash, and be applied to the foot.

**Conclusions:**

To the extent possible, MAP developers should consider method designs and formulations that limit menstrual side effects and provide more than one month of protection.

## Plain english summary

Existing contraceptive methods do not meet the needs and preferences of all users. New technologies, particularly ones that are user-controlled and do not require administration by a healthcare provider, may increase use and reduce unintended pregnancies. Efforts are underway to develop a microarray patch (MAP) for contraception, which would be a potentially painless, self-administered product. We conducted research in India and Nigeria to determine what characteristics of this new method potential users would like or not like to inform the design of the product. We conducted a discrete choice experiment survey, which involved asking study participants to choose between several sets of hypothetical products with six varying attributes: effect on menstruation, application pain, location of application, rash after application, patch size, and duration of pregnancy prevention. The results indicate that women in both countries were most interested in a product that would not affect menstruation compared to one that would cause irregular bleeding or that would stop their period. The second most important characteristic was duration of effectiveness – most women preferred a product that lasted six months rather than three months or one month. MAP developers should consider users’ preferences for no menstrual side effects and a longer-acting product when making design decisions to ensure the development of a successful product.

## Background

In low- and middle-income countries (LMICs), an estimated one in three women who would like to avoid or delay pregnancy are not using a modern method of contraception [1]. Reasons documented for this unmet need for family planning (FP) include problems with access to FP methods, opposition to contraception from women and/or their sexual partners, and method-related reasons [1, 2]. Across 52 Demographic and Health Surveys, the most common reason married women gave for not using a method was side effects/health concerns related to FP methods [3]. New contraceptive technologies have the potential to overcome method-related reasons for non-use and should be part of a multi-pronged strategy to reduce unmet need [4].

New technologies that expand women’s contraceptive options also present an opportunity to promote self-care, which the Lancet Global Health Commission recently described as vital to the future of quality healthcare by shifting power from the healthcare system and providers to patients [5]. Self-care options allow individuals, families, and communities to manage and promote their own well-being with or without the support of a healthcare worker. In the realm of family planning, condoms, pills, and emergency contraceptives have offered “self-care” options for decades, and new delivery methods such as self-injection with Sayana Press® [6], and still in early stages of development, a microarray patch (MAP) have the potential to give women even more options under their control.

MAPs are small patches containing hundreds of microneedles that are applied to the skin to deliver a drug or other therapeutic [7, 8]. MAPs offer the potential for a self-administered product with simplified distribution and storage and no sharps waste. Recently, efforts to develop a MAP to deliver contraceptive steroids have shown promise [9, 10]. To optimize the design features of this innovative product and ensure its eventual success, it is important to incorporate the perspectives of potential end users.

This paper describes the results of a sequential, exploratory study designed to examine potential acceptability of a contraceptive MAP among prospective end-users, define desired attributes of the product, and quantify the relative importance of particular method characteristics. The study included an initial qualitative component [11], results of which were used to inform the design of a discrete choice experiment (DCE) survey presented in this paper. DCEs are a stated preference method used to assess the effect that specific product characteristics have on consumer choice. DCEs have gained popularity in the field of public health over the past decade, though applications to contraceptive preferences remain limited and tend to address generic preferences rather than inform the design of a specific product [12, 13]. DCE survey respondents are presented with a series of hypothetical scenarios and asked to choose which they prefer. Each scenario is described in terms of several characteristics (called attributes) that vary in their levels. Response data are analyzed to estimate how much each attribute and level influence respondents’ choice of scenario. DCEs can be particularly useful to understand preferences when it is difficult or not possible to observe actual choices and are relevant to inform the design of new products (like the MAP) and interventions that do not yet exist in the market [14–18].

We conducted this study in two LMIC settings, New Delhi, India and Ibadan, Nigeria, which represent contraceptive markets with varying contraceptive prevalence and method availability. Modern contraceptive prevalence in India is 47.8% with most women using permanent contraception [19]. In Nigeria 12% of married women and 28% of sexually active unmarried women currently use modern contraception, most commonly contraceptive implants and condoms [20]. The objective of the DCE described here was to determine the importance of a set of MAP characteristics relative to each other for potential users in these settings to inform product design decisions.

## Methods

### Development of the Discrete Choice Experiment (DCE) Survey

Attribute development and level selection are cornerstones of DCE design, as misspecification can lead to biased or useless results. We based the development of the DCE on an initial qualitative component of this study including focus group discussions (FGDs) and in-depth interviews (IDIs) with women and IDIs with family planning providers. The methods and results of this qualitative phase are presented elsewhere [11].

**Fig. 1:**
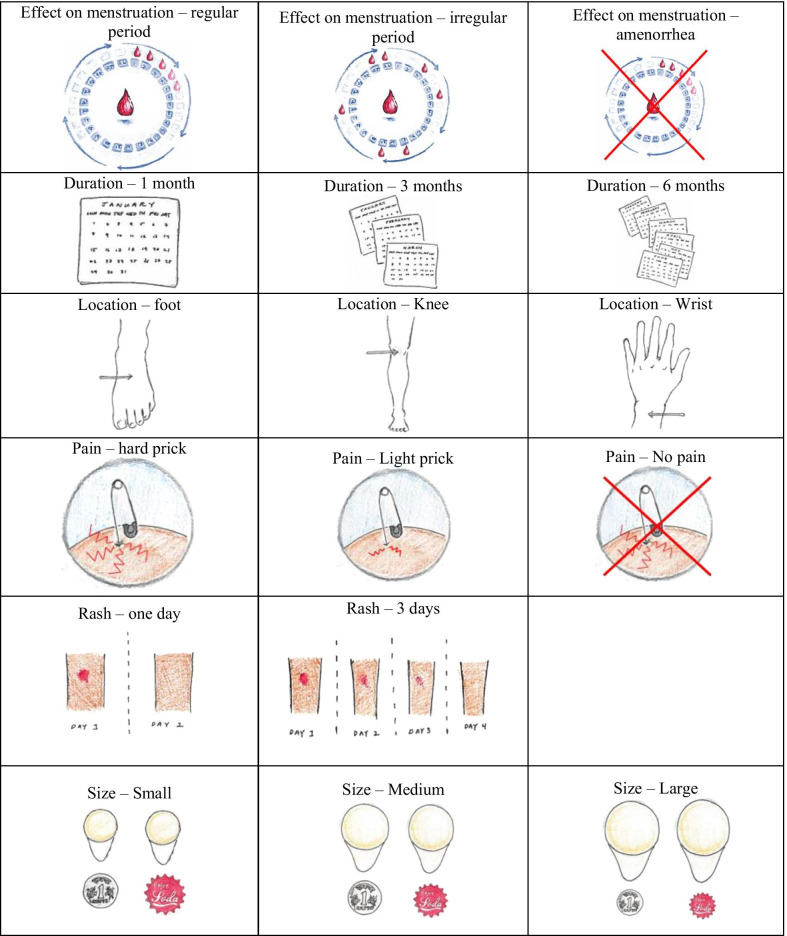
Attributes and levels included in the discrete choice experiment. *Version used in Nigeria included darker skin tone for pain and rash illustrations. One rupee coin used for the size illustration in the India survey and the soda top in the Nigeria survey

We selected attributes and levels for the DCE separately for each country, first for India and later for Nigeria. For India, three team members independently reviewed analytical memos summarizing findings for attributes and levels discussed in the qualitative interviews. Next, the team compiled a reduced and prioritized list of attributes and levels based on their relevance for the qualitative interview participants, technical plausibility for a MAP, and methodological considerations limiting the number of attributes and levels that can be included in a DCE. The reduced list was then reviewed with the lead product developer at the Georgia Institute of Technology to determine whether the selected attributes and levels were realistic, credible, and pertinent to inform design decisions for the MAP. A similar process was used in Nigeria, although the memos reviewed as an initial step only covered half of the transcripts since findings were similar to those from India. Method side effects were not originally included as an attribute in the qualitative phase; however, frequent spontaneous discussions combined with technical considerations related to the implications of different possible hormonal formulations (combined estrogen and progestin or progestin-only) for bleeding patterns prompted us to add an attribute related to effects of MAP use on menstruation. The levels (wrist, kneecap, and top of foot) for the location of application attribute were suggested by the product developer because they offer a “harder” surface compared with other parts of the body, which may be important for more complete separation of the microneedles from the patch backing. The full list of attributes and levels included in the DCE is shown in Fig. 1.

### Additional insights from cognitive interviews

Prior to implementation of the DCE survey in each country, two local consultants each paired with a study investigator conducted iterative rounds of cognitive interviews (CIs) with a convenience sample of women. The CIs tested clarity of wording in local languages, assessed comprehensibility and appropriateness of the attributes and levels and illustrative pictures, and checked participant understanding of the DCE task. Ten CIs were conducted across three rounds in India, and eight CIs across two rounds in Nigeria. Changes were made between rounds based on feedback received; changes were minimal by the final round in each country, indicating that saturation had been reached.

### Eligibility and sampling for the DCE survey

Data collection occurred in November 2017 in India and between March–April 2018 in Nigeria. The DCE survey implemented in India contained all six attributes. Due to concerns that one of the attributes (effect on menstruation) may be dominant, two versions of the surveys were fielded in Nigeria: one with all six attributes and one with five, removing effect on menstruation.

In India, women were eligible for the study if they were married and aged 15–49. In Nigeria, both married women aged 15–49 and unmarried women aged 18–49 were eligible if they reported being sexually active in the last 30 days. Unmarried women were not included in India due to the more conservative cultural context, which made accessing this population challenging. Because women who have had experience using modern contraception may have different perspectives on desired method characteristics than women who have never used contraception, we stratified our sample by ever use of contraception. In India, due to the high prevalence of female sterilization, sterilized women were categorized as ever or never user based on contraceptive use prior to being sterilized. Women currently not using modern contraception in both settings were eligible if they reported not being opposed to contraceptive use.

Women were selected through a multi-stage random sampling process. Ten census enumeration blocks divided between urban and peri-urban were selected from three municipal corporations in and around Delhi in India (East, North and South Delhi). In Nigeria, 10 enumeration areas were randomly selected to include five from an urban locality (Agbowo in the Local Government Area (LGA) of Ibadan North) and five from a peri-urban locality (Ajibode in the Akinyele LGA). Being urban and peri-urban, these areas included a range of dwellings including single and multi-family homes, both free-standing and apartments. All households and eligible women within the selected areas were listed. Research assistants individually screened each woman to ascertain eligibility and stratum of inclusion. Women were randomly selected within each population subgroup; no more than one eligible woman per household was selected. Up to three attempts were made to contact each sampled woman.

### Sample size

Despite some recent efforts (see for example, de Bekker-Grob et al., 2015), no consensus yet exists on the best way to estimate the sample size required for a DCE for obtaining meaningful, statistically robust parameter estimates given the multiple parameters and comparisons of interest [21]. For our purposes, we considered the popular rule of thumb of Johnson and Orme.

$$N \, > \, 500c/\left( {t \, x \, a} \right),$$

where *c* is the largest number of levels for any of the attributes, *t* is the number of choices sets to be given a respondent, and *a* is the number of options within choice sets [22, 23]. Orme [24] cautioned, however, that the rule of thumb was intended as a minimum and recommended that researchers try to at least double this minimum sample size.

For this study, the largest number of levels for any attribute, *c*, was 3; the initial number of choice sets to be given a respondent, *t*, was 10; and the number of options within each choice set, *a*, was 2 resulting in a sample greater than 500*3/(10*2), or a minimum of 75 for each population group in each country. In India, we increased this minimum requirement to 125 women in each stratum (contraceptive use, urban/peri-urban setting), for a total of 500 women. In Nigeria, where we implemented two versions of the survey, given resource constraints and the addition of a third stratum (marital status), we aimed for 130 women for sample 1 (six attributes) and 100 for sample 2 (five attributes) for any population group defined by two stratifying variables.

### Data collection

All DCE surveys were conducted with women in their homes by trained data collectors in the local language. Written consent was obtained from all participants. Sigma Institutional Review Board in India, the Oyo State Research Ethical Review Committee in Nigeria, and FHI 360′s Protection of Human Subjects Committee in the United States approved this study.

We used Sawtooth Software Lighthouse Studio v.9.5.2 to program and administer the DCE via handheld tablets. Respondents were presented 10 sets of two MAP designs. An additional fixed pair was presented to the respondent at the start of the survey to ensure comprehension of the choice process. Choice pairs were randomly generated to have balanced overlap between attribute levels. The efficiency of the DCE design was tested using the “test design” feature in Lighthouse Studio. We checked the estimated main effects and their standard errors in the test run to assess whether the design provides reasonable precision for the model estimates. We also checked the D-efficiency of the design, a commonly used metric to assess statistical efficiency.

### Statistical analysis

Data were uploaded from the study tablets into Sawtooth Software’s Lighthouse Studio for analysis. The fixed choice set was not included in the analysis. We used Choice-based Conjoint with Hierarchical Bayes estimation to calculate individual utilities for each attribute level within each participant. Utilities used to estimate the model parameters are averages based on the frequency of choosing a contraceptive MAP with the given attribute level. The final model coefficient estimates are obtained in an iterative process using a Monte Carlo Markov Chain process and used to represent the utility of each attribute level for the population. We also examined first-order interactions between attributes. Interactions were included in the final model if they were statistically significant at the level of *p* ≤ 0.05, made intuitive sense, and improved the model fit. We also assessed associations between sociodemographic covariates including age, education level, and prior use of a modern method and attribute preferences and included in the final model if statistically significant defined as *p* ≤ 0.05. All models included age, education, and ever use of modern method use as covariates.

Using the final model, we obtained individual utilities for each attribute level within each participant. We then characterized the relative importance of each attribute by considering how much difference each attribute could make in the total utility of a product. The greater the difference, the greater the impact that an attribute could make in choosing a product. That difference is the range in the attribute’s utility values (e.g., a difference of 30 from a minimum utility of 10–40). These differences are computed for each attribute and the relative importance is the percentage of each attribute’s utility difference relative to the total utility range (i.e., sum of all attribute’s utility differences). These relative importance values are obtained for each individual and the final estimate of relative importance for each attribute is obtained by averaging these values across the entire sample.

Another way of using utility estimates that may be easier to interpret is by using market simulations. Using the final model, we computed the proportion of potential users in the three samples who would choose hypothetical MAP products using the market simulation function in Lighthouse. To demonstrate the potential gain in user desirability with changes to the MAP design, each hypothetical MAP product was compared with a reference MAP with the following attribute levels: a medium sized patch providing three months of pregnancy protection that would be applied to the wrist with pain similar to a light pin prick resulting in a three-day rash and an irregular period.

## Results

**Fig. 2:**
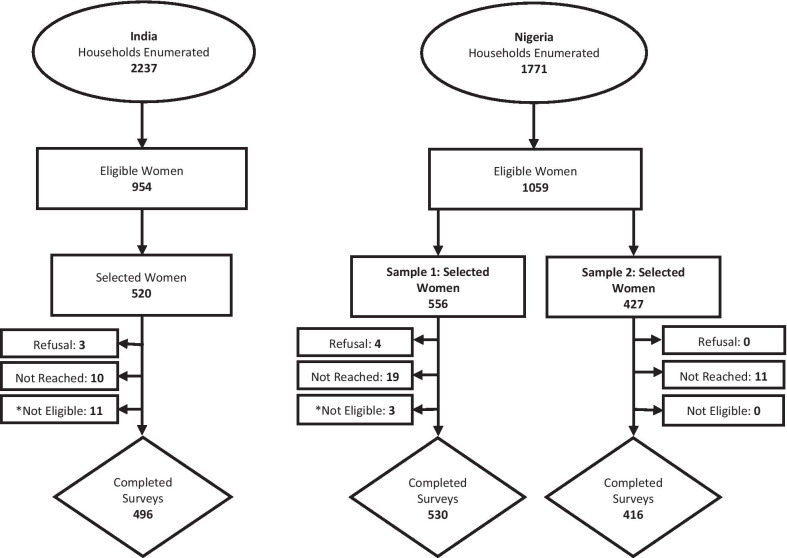
Sample selection for the discrete choice experiment in India and Nigeria. *Determined to be not eligible after being reached for interview

Survey response rates were 97% in India and 95% (sample 1) and 96% (sample 2) in Nigeria. A total of 496 women in India and 530 women in Nigeria (sample 1) completed the DCE with six attributes and 416 women in Nigeria completed the version with 5 attributes (sample 2) (Fig. 2). Socio-demographic characteristics of each sample are summarized in Table 1. Women in India and Nigeria had mean ages of 33 and 29, respectively. About 70% of Indian respondents were currently using a method of contraception, with about half reporting being sterilized. In Nigeria slightly more than a third were using a method, the most common being condoms. Most respondents in India did not want more children (79%), while three-quarters of Nigerian women reported desiring more. Education was overall lower in India than in both Nigerian samples, with more than half of women in the Nigerian samples completing higher education.

**Table 1:** Sociodemographic characteristics of potential contraceptive microneedle patch users in India and Nigeria

	India (N = 496)	Nigeria Sample 1 (N = 530)	Nigeria Sample 2 (N = 416)
	%		
Age (mean, SD)	33.0 (7.3)	28.8 (7.5)	29.3 (7.7)
Current contraceptive status			
Sterilized, has used FP	5.2	–	0.2
Sterilized, never used FP	27.6	0.2	0.5
IUD	13.1	2.1	3.4
Implant	–	2.8	4.8
Injectable	0.8	4.7	4.6
Pill	4.0	2.1	3.1
Condoms	17.9	20.8	14.9
EC	–	3.4	6.7
Traditional method	1.4	20.8	9.1
Non-user, has used FP	8.1	12.1	11.5
Non-user, never used FP	21.8	31.1	41.1
Level of education			
No education	14.7	1.1	0.5
Some primary	7.1	0.8	0.5
Completed primary	13.7	6.8	4.6
Some secondary	11.9	5.8	7.5
Completed secondary	19.2	26.0	24.3
Higher	33.5	59.4	62.7
Religion			
Hindu	93.3		
Christian	0.8	68.7	68.5
Muslim	5.8	31.3	31.3
Number of children (mean, SD)	2.5 (1.1)	1.3 (1.6)	1.4 (1.6)
Desire for more children			
Want more	12.9	76.0	75.5
Do not want any more	79.2	16.8	18.3
Undecided	4.8	0.1	6.3

**Fig. 3:**
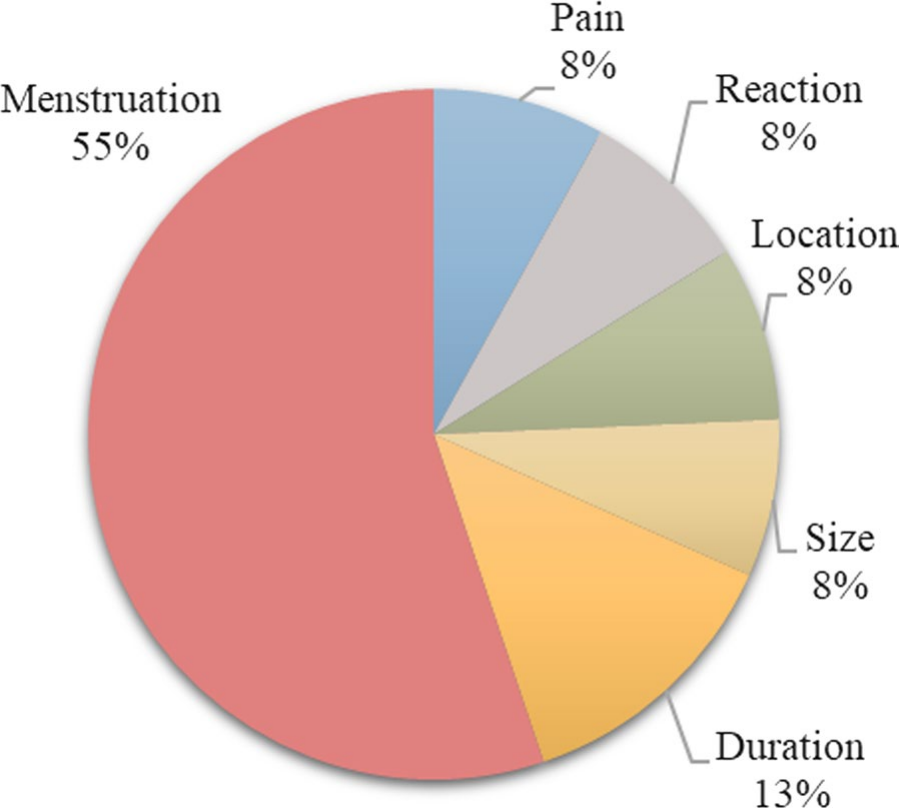
Relative importance of MAP attributes, India (N = 496)

**Fig. 4:**
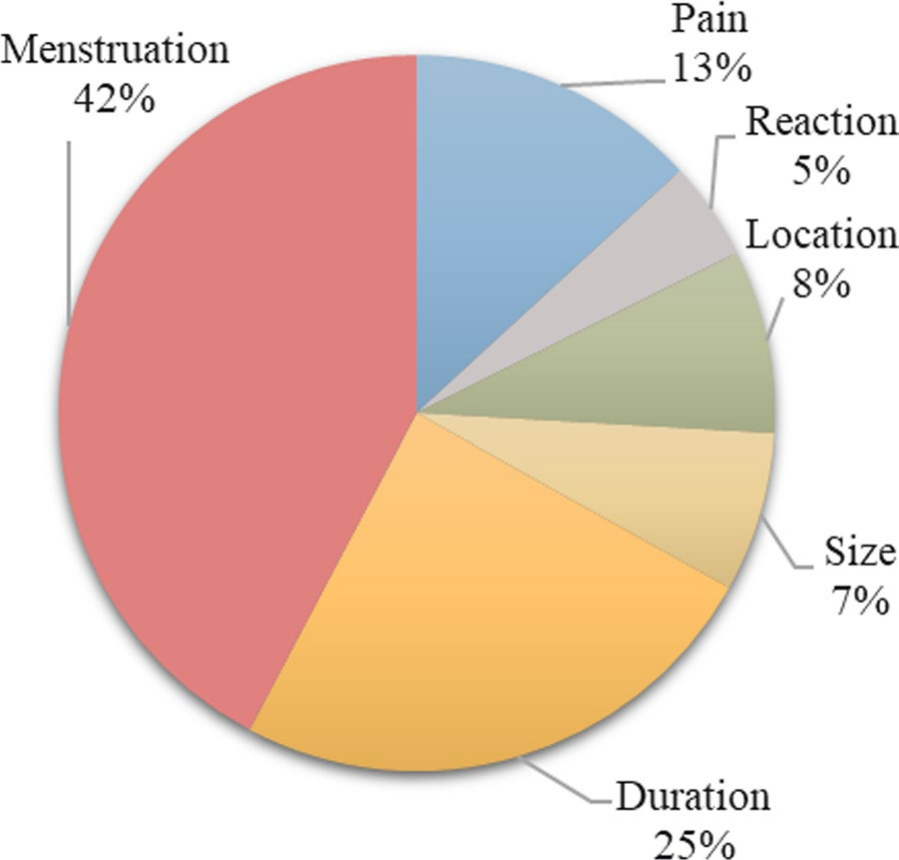
Relative importance of MAP attributes, Nigeria Sample 1 (N = 530)

**Fig. 5:**
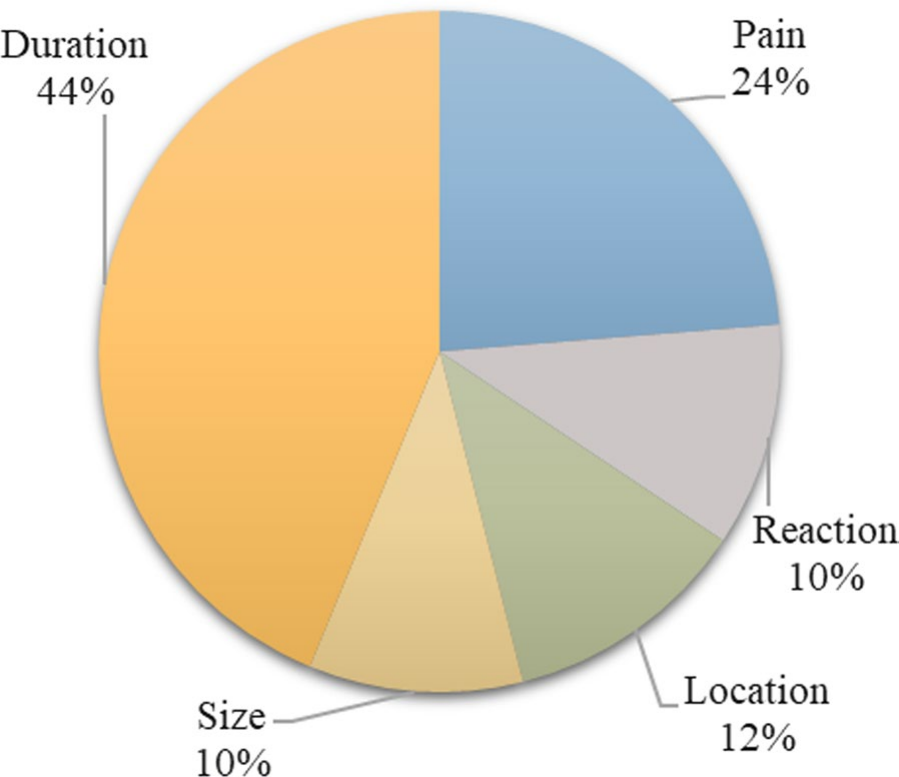
Relative importance of MAP attributes, Nigeria Sample 2 (N = 416)

**Fig. 6:**
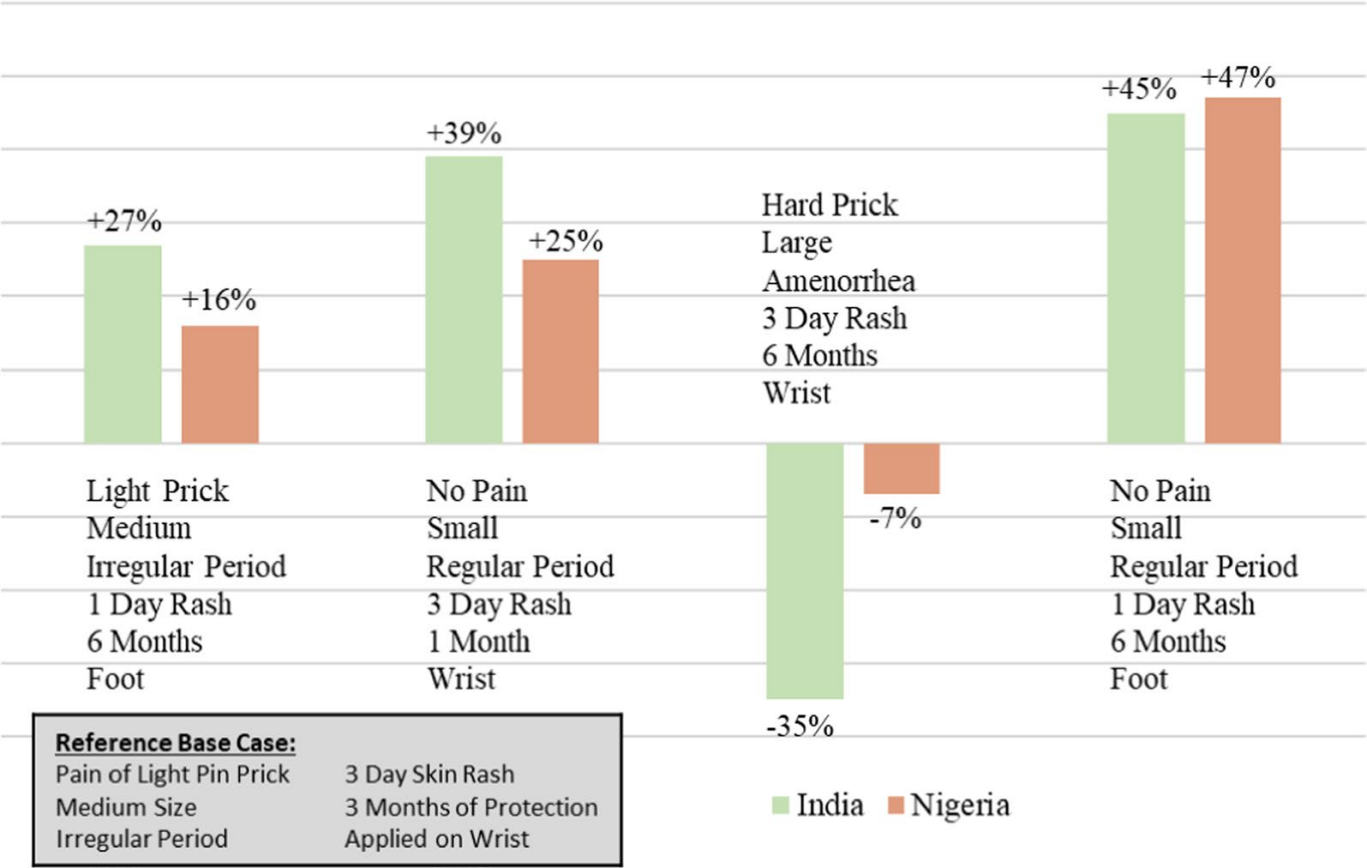
Simulations of change in preference with different hypothetical contraceptive MAP designs

Figures 3, 4, 5 and 6 display the relative importance of each MAP attribute included in the DCE, i.e., how much relative influence that attribute would have on the total utility of a MAP product, based on the range in the attribute’s utility values (utility estimates are shown in Table 2). Effect on menstruation was four times more important for Indian women (55% relative importance (95% CI 53.6–56.7)) and two and a half times more important for Nigerian women (42% relative importance (95% CI 40.7–43.8)) than duration of effectiveness, which was the second most important attribute in both settings. The remaining attributes had similarly low utilities in both countries. In Nigeria Sample 2 where the effect on menstruation attribute was not included in the DCE, duration emerged as the most important choice driver with approximately double the importance of the next most important attribute, pain.

**Table 2:** Utility estimates from Hierarchical Bayes models

	HB Model 1: India				HB Model 2: Nigeria				HB Model 3: Nigeria		
Attribute levels	*B*	SD	95% CI		*B*	SD	95% CI		*B*	SD	95% CI
No pain at application	13.0	23.0	(11.0, 15.0)		33.0	32.2	(30.2, 35.7)		58.5	38.0	(54.9, 62.2)
Light prick	3.8	22.1	(1.8, 5.7)		− 1.9	24.5	(− 4.0, 0.2)		− 4.4	20.3	(− 6.4, − 2.5)
Hard prick	− 16.8	24.2	(− 18.9, − 14.6)		− 31.0	30.0	(− 33.6, − 28.5)		− 54.1	37.4	(− 57.7, − 50.5)
One day rash	20.8	20.6	(19.0, 22.6)		10.7	12.6	(9.7, 11.8)		25.2	20.8	(23.2, 27.2)
Three day rash	− 20.8	20.6	(− 22.6, − 19.0)		− 10.7	12.6	(− 11.8, − 9.7)		− 25.2	20.8	(− 27.2, − 23.2)
Foot application	4.1	20.4	(2.3, 5.9)		− 9.1	19.2	(− 10.8, − 7.5)		− 12.7	30.4	(− 15.7, − 9.8)
Knee application	− 10.6	25.2	(− 12.8, − 8.4)		− 0.5	28.2	(− 2.9, 1.9)		12.1	31.3	(9.1, 15.1)
Wrist application	6.5	25.9	(4.2, 8.8)		9.7	24.1	(7.6, 11.7)		0.7	27.0	(− 1.9, 3.3)
Small size	7.5	25.6	(5.2, 9.7)		8.1	20.5	(6.4, 9.9)		17.3	25.0	(14.8, 19.7)
Medium size	0.2	18.3	(− 1.5, 1.8)		6.0	16.9	(4.6, 7.5)		3.7	13.1	(2.4, 4.9)
Large size	− 7.6	21.9	(− 9.6, − 5.7)		− 14.2	23.4	(− 16.2, − 12.2)		− 20.9	27.4	(− 23.5, − 18.3)
One month duration	− 30.3	38.7	(− 33.7, − 26.9)		− 59.9	73.5	(− 66.2, − 53.7)		− 96.8	82.8	(− 104.8, − 88.9)
Three month duration	0.7	20.4	(− 1.1, 2.5)		11.3	18.8	(9.7, 12.8)		13.5	18.8	(11.7, 15.3)
Six month duration	29.7	30.5	(27.0, 32.3)		48.7	66.4	(43.0, 54.3)		83.3	78.1	(75.8, 90.8)
Regular menses	177.0	84.7	(169.5, 184.4)		114.3	80.8	(107.4, 121.2)				
Irregular menses	− 45.9	39.3	(− 49.4, − 42.4)		− 52.0	65.0	(− 57.6, − 46.5)				
Amenorrhea	− 131.1	71.1	(− 137.3, − 124.8)		− 62.3	101.3	(− 70.9, − 53.7)				
Interactions											
1 mo x Regular	− 41.2	26.3	(− 43.5, − 38.9)								
1 mo x Irregular	7.2	15.9	(5.8, 8.6)								
1 mo x Amenorrhea	34.0	33.7	(31.0, 37.0)								
3 mo x Regular	1.5	15.8	(0.1, 2.9)								
3 mo x Irregular	3.4	18.4	(1.8, 5.0)								
3 mo x Amenorrhea	− 4.9	16.8	(− 6.4, − 3.4)								
6 mo x Regular	39.7	25.0	(37.5, 41.9)								
6 mo x Irregular	− 10.6	20.9	(− 12.4, − 8.8)								
6 mo x Amenorrhea	− 29.1	27.1	(− 31.4, − 26.7)								
Model statistics											
Percent certainty	0.7				0.7				0.7		
Fit statistic (RLH)	0.8				0.8				0.8		
Covariates	3				3				3		

*B* are estimated model coefficients representing the utility of each attribute level for the population. *B*, SD and 95% CI are obtained in an iterative process using a Monte Carlo Markov Chain process

The HB model utility estimates shown in Table 2 indicate that women in both countries preferred to maintain a regular period over the prospect of irregular menstruation or amenorrhea with the latter being particularly unappealing in India. Respondents also preferred a six-month product to one that would last three or one month. In the India sample, duration interacted with the effect on menstruation: a product that would last for one month and cause amenorrhea had a positive effect on product choice in India while longer durations had a negative effect on choice when combined with irregular menstruation or amenorrhea. In both countries, pain described as a “hard pin prick” was a choice deterrent; however, the relative utility estimate was stronger for Nigerian women. Location of application preferences also varied between the two countries, with Indian respondents preferring the wrist or foot more than the kneecap and Nigerian respondents not preferring application on the foot. Other than a slightly higher tolerance for bleeding changes among women who had used contraception in the past, we did not see substantial variation in attribute preferences between ever and never users of contraception or among women of different ages or education level in either country (results not shown).

Results of the choice simulations are shown in Fig. 5. The most ideal design would be a small patch, providing six months of protection, that would involve no pain on administration, result in a one- day rash, and be applied to the foot. Such a design would be chosen nearly 50% more frequently in both countries compared to the reference design of a medium sized, three-month patch applied on the wrist with light pain resulting in a three-day rash and potential for irregular menstruation.

## Discussion

This paper describes results from a discrete choice experiment examining potential users’ preferences for the design of a contraceptive MAP in Delhi, India and Ibadan, Nigeria. For women in both contexts, the effect that a contraceptive MAP would have on their menstruation was most important in their choice between two hypothetical products with other varying characteristics. In both countries, women also preferred a six-month product over one that would last three or one month, with the latter being the least preferred duration. In India, an interaction between duration and menstruation may indicate that women would be more accepting of a one-month product that causes amenorrhea; however, how women understood the duration of amenorrhea in this scenario is unknown. It could be that women felt more comfortable inducing amenorrhea with a short-acting method that they could more rapidly discontinue. More research is needed to understand the nuances of women’s preferences for and acceptability of contraceptive-induced menstrual changes and amenorrhea, in particular. Perhaps not surprisingly, women who had prior experience using modern contraception were more likely to accept a product that would alter their period than women who hadn’t used a modern method; however, both groups would prefer to maintain a regular period.

A recent scoping review comprising 100 studies with data on women’s responses to contraceptive-induced menstrual bleeding changes concluded that menstrual changes are top reasons for method dissatisfaction, discontinuation, and non-use [25]. While the authors point out that substantial variability exists around responses to bleeding changes based on individual experience and social influence, the importance of contraceptive-induced bleeding changes for women’s daily lives and contraceptive satisfaction have been underappreciated by family planning programs and contraceptive product developers. Our results support the observation that menstrual side effects are top of mind for women considering their contraceptive options; both irregular menstruation and amenorrhea were less appealing to women in this study than maintaining a regular period. Current MAP development efforts, however, are focused on progestin-only formulations which will inevitably cause menstrual changes. While a progestin-only approach is appropriate for a safe and long-acting (e.g., six month) MAP, product developers might consider a combined estrogen-progestin formulation that would be shorter-acting but offer the potential for maintaining regular menstruation.

The strong importance of the menstruation attribute for women’s stated product preference makes it somewhat difficult to interpret the importance of the other attributes included in the DCE. While duration of protection and application pain emerged as the second and third most important characteristic for women, respectively, the importance of skin reaction duration, application location, and patch size less clearly influenced choice. Our findings in the preceding qualitative phase of this study demonstrate that these attributes are important particularly as they relate to women’s desire for a product that may be used discreetly in a location that they can observe [11]. The locations presented as attribute levels in the DCE (wrist, kneecap, and foot) were selected for application feasibility but were not particularly well received by potential users. MAP developers should further consider where on the body the MAP could be applied since MAP choice may hinge on the user’s ability to hide any skin reaction the product causes.

### Limitations

DCEs are useful tools for measuring the relative importance of a set of predetermined product characteristics; however, they are inherently limited by the attributes and levels included in the choice sets. The number of attributes and levels is further limited by how many different characteristics respondents can consider at once. While the attributes and levels we included in the DCE were based on strong qualitative data, we cannot rule out that other product characteristics may be more important for potential users. This study was designed to examine stated preferences for MAP attribute levels, not the choice between a MAP and other existing contraceptive products on the market. Therefore, it does not provide information on whether women may potentially prefer this option to other products. Future research will be needed to understand the product’s potential market share or women’s preference for a MAP over other existing options. Finally, while our study included a strong sampling design and we believe that our findings can be generalized to women living in urban and peri-urban areas of southern Nigeria and northern India, they are not representative of women’s preferences globally.

## Conclusion

The incorporation of user preferences is key for designing products that people want to use. This is especially true for novel products such as a contraceptive MAP, which is unlike any currently available method. MAPs offer potential for a user-controlled method that could expand contraceptive self-care to women around the world. We have found that women are excited about this method [11], with a preference for a product that would offer several months of pregnancy protection while not having menstrual side effects. While it may not be possible to create a product that satisfies both of these desires, product developers should consider exploring both single and combined steroid MAP formulations that would give users even more contraceptive choice.

## Data Availability

The datasets generated and/or analysed during the current study are available in the Harvard Dataverse repository, https://dataverse.harvard.edu/dataset.xhtml?persistentId=doi%3A10.7910%2FDVN%2FTOSSWN&showIngestSuccess=true&version=DRAFT#

## Abbreviations

CI: Cognitive interview
CORT: Centre for Operations Research and Training
DCE: Discrete choice experiment
FP: Family planning
FGD: Focus group discussion
IDI: In-depth interview
LMIC: Low- and middle-income countries
LGA: Local Government Area
MAP: Microarray patcH
